# Root Plasticity in the Pursuit of Water

**DOI:** 10.3390/plants8070236

**Published:** 2019-07-22

**Authors:** Hillel Fromm

**Affiliations:** School of Plant Sciences and Food Security, Faculty of Life Sciences, Tel Aviv University, Tel Aviv 69978, Israel; hillelf@tauex.tau.ac.il

**Keywords:** drought, hydraulic lift, hydropatterning, hydrotropism, phenotypic plasticity, rhizosphere, root system architecture, xerobranching, xerotropism

## Abstract

One of the greatest challenges of terrestrial vegetation is to acquire water through soil-grown roots. Owing to the scarcity of high-quality water in the soil and the environment’s spatial heterogeneity and temporal variability, ranging from extreme flooding to drought, roots have evolutionarily acquired tremendous plasticity regarding their geometric arrangement of individual roots and their three-dimensional organization within the soil. Water deficiency has also become an increasing threat to agriculture and dryland ecosystems due to climate change. As a result, roots have become important targets for genetic selection and modification in an effort to improve crop resilience under water-limiting conditions. This review addresses root plasticity from different angles: Their structures and geometry in response to the environment, potential genetic control of root traits suitable for water-limiting conditions, and contemporary and future studies of the principles underlying root plasticity post-Darwin’s ‘root-brain’ hypothesis. Our increasing knowledge of different disciplines of plant sciences and agriculture should contribute to a sustainable management of natural and agricultural ecosystems for the future of mankind.

## 1. Introduction: The Challenge of Water Acquisition and Root Phenotypic Plasticity

As plants colonized the land hundreds of million years ago, replenishing their declining water supply has become one of their greatest challenges. Water deficiency has also become one of the most detrimental environmental stresses in agricultural and ecological systems, exacerbated by increasing climate change [1,2]. On a global scale, climate variation explains a third of crop yield variability [3]. In substantial areas, for example in parts of Western Australia, over 60% of wheat yield variability can be explained by climate variability [3]. In this context, water shortages are responsible for the greatest crop losses around the world [4]. Within the U.S. alone, approximately two thirds of all crop losses in the last 50 years have been due to drought [4], and more frequent occurrences of water shortages are expected due to climate projections and increasing competition for water among urban, industrial, and agricultural demand [4]. Drought also causes reduction in global terrestrial net primary production, which is the amount of atmospheric carbon fixed by plants and accumulated as biomass [5]. A continued decline in net primary production is expected to weaken the terrestrial carbon sink and to intensify competition between food demand and biofuel production [5].

To mitigate episodes of water deficiency, plants have evolutionarily acquired different developmental and defense responses, which are often classified as: (i) ‘Escape’, which consists of environmentally regulated developmental programming to evade the stress, such as seasonal-dependent germination (regulated by day length), stimulus-dependent germination (regulated by soil moisture or temperature), flowering time (to avoid terminal drought); (ii) ‘Avoidance’, which includes developmental, morphological, and physiological adaptations to minimize the effects of an existing stress, such as osmotic adjustments [6], stomata aperture control to maintain leaf water potential [7], reducing stomata density [8,9], and changes in root architecture and traits; and (iii) ‘Tolerance’, which is the ability to survive a stressful situation while maintaining basic biological processes. An extreme example is abscisic acid (ABA) signaling in resurrection plants [10]. Such survival mechanisms come into action when drought become too severe, and drought avoidance mechanisms are no longer sufficient [11]. The physiological integrity of a plant is maintained if avoidance and tolerance mechanisms are sufficient to prevent damage [11].

Phenotypic plasticity is defined as the ability of an organism to change its phenotype in response to environmental conditions [12,13]. To better understand root plasticity, first, it is necessary to view the structure of the root system, or as it is most commonly referred to, the Root System Architecture (RSA), defined as the geometric arrangement of individual roots within the plant root system in the three-dimensional soil space [14,15,16]. Both monocotyledon (monocot) and dicotyledon (dicot) root systems consist of embryonically derived primary roots (the radicle), lateral roots that branch from primary roots, and further branching of lateral roots from previously formed lateral roots. Furthermore, both monocots and dicots may develop adventitious roots from non-root tissues (e.g., from the scutellum in monocots, and from lower underground stem nodes in both monocots and dicots). The maize root system consists of a primary root that develops from the radicle, seminal roots that branch from the scutellar node, crown roots, which are roots post-embryonically derived from the lowermost belowground nodes of the stem, and brace roots that develop from above-ground stem nodes. The primary and seminal roots are highly branched and fibrous. Similar fibrous root systems are found in other cereals such as wheat and rice [15,16]. The crown roots are relatively unimportant in younger seedlings, in contrast to primary and seminal roots. However, crown roots continue to branch and develop throughout the vegetative stage and they sometimes comprise the major part of the root system. The root system of dicots consists of a primary (tap) root and its branch roots. Basal roots may arise from the base of the tap root and in addition, adventitious roots may develop from the stem and hypocotyl, analogous to the crown roots in cereals. The ability of roots to develop from non-root tissues gives them tremendous flexibility in a wide range of habitats for improving water and mineral acquisition and to serve as physical support (particularly in trees). RSA varies widely among species, even among those living in the same habitat and at the same time. It also varies tremendously within the same species, depending on the underground and aboveground conditions, with great plasticity for resource acquisition, including water [17].

Typical root development includes the formation of root hairs, which are extensions of epidermal cells. This development occurs in a defined zone of the root behind the elongation zone. Root hairs (and rhizosheath) greatly increase root-soil contact and the surface area available for adsorbing water and nutrients [18] and for plant-microbe interactions [19]. In many plant species, every root epidermal cell has the potential to differentiate into a root hair. In other species (including *Arabidopsis*) the root epidermis consists of alternating groups of cells that are either atrichoblats (cannot form root hairs) or trichoblasts (can form root hairs). *Arabidopsis* mutants lacking root hairs are highly vulnerable to drought stress and exhibit reduction in water absorption [20]. A similar response was observed in root-hairless mutants of barley, which exhibited a higher susceptibility to drought stress than did their parent cultivar [21]. In common bean, it was demonstrated that a longer root hair phenotype has superior biomass accumulation (89%) compared with a short root hair phenotype under phosphorous stress conditions [22]. Thus, root hairs are important as environmental sensors and for acquiring nutrients and water.

To facilitate research toward crop improvement, scientists have suggested describing the phenotype of roots in sub-phenotypic elements termed phenes [23]. A phene is an element of the phenotype; it is analogous to a gene being an element of the genotype. Phene states represent the variation in form and function of a particular phene [23]. For breeding purposes, phenes may consist of any trait that contributes to root function: Developmental, anatomical, metabolic, and physiological. Phenes that influence the same functions (e.g., specific nutrient acquisition or utilization) most likely operate within a phene module. Better understanding how root phenes interact to affect soil resource acquisition will serve as an important tool in breeding crops with superior stress tolerance and reduced dependence on inputs. For example, root hairs and larger diameter root tips in cereals were associated with water acquisition from drying soils. These anatomical phenes could be targets for crop improvement [24]. Similarly, maize phenes associated with nitrogen and phosphorus acquisition have also been identified [25,26].

Phenes can be further assembled into a broader characteristic ideotype, regarded as a biological model expected to perform and behave in a specific manner within a defined environment. In particular, the ideotype is expected to yield a greater quantity and quality when developed as a cultivar [27]. For example, an ideotype consisting of specific phenes that may contribute to rooting depth in maize under water-limiting conditions has been described [28]—for example, a large diameter of primary root with long laterals and long root hairs. [28]. On the other hand, breeding crops for irrigated agricultural systems requires defining other ideotypes that are more efficient in water and nutrient acquisition from shallow strata [27]. 

From an ecological perspective, increasing evidence over recent years suggest that root traits may explain a range of ecosystem properties better than shoot traits [29]. For example, root traits have been found to explain soil microbial community composition, availability of nitrogen, rates of nitrification, and plant performance at a population level [29]. 

## 2. Root Phenotyping: In Vitro, in Soil, and in the Field

In the past few years, we have witnessed impressive progress in our ability to monitor root development in soil-grown plants, mainly by using instruments that were originally designed for medical diagnostics. These include MRI and X-ray computer tomography [30]. These technologies enable one to characterize root developmental patterns in soil, in response to the distribution of various resources including phosphate and water [30,31,32,33]. Furthermore, these technologies facilitate characterizing gene functions while determining precise root growth patterns, including the angle of roots [34] and the emergence of lateral roots [31,32,35]. Such technologies have also been used for determining water content and distribution in the vicinity of the root growing in mini-lysimeters by fitting their size to that of the MRI transmitter coil [36], or by using X-ray computer tomography with image-based modeling to detect plant water uptake in soil columns [37,38]. However, none of these technologies are suitable for field studies. Indeed, one of the greatest challenges in plant phenotyping is field-based root phenotyping in natural ecosystems and in crops [39,40]. Nevertheless, progress is being made in developing imaging technologies and computational tools (modeling and in-depth learning) for field-based root phenotyping [41,42,43]. These innovations are being integrated into broader, multiscale models of plant behavior, ranging from molecules, through cells, organs, to whole plants and ecosystems [44,45]. The holistic models of crops in the context of their ecosystems are important for developing sustainable agriculture in a world that increasingly requires a substantial increase in food production while facing the detrimental effects of climate change. Other established methodologies, like aeroponics, provide certain advantages for studying specific aspects of root biology [46,47,48]. 

Although these approaches have greatly enhanced our understanding of root development and responses to the environment, further understanding of root behavior requires detecting cellular and inter-cellular signals in the root and their spatial and temporal dynamics. To date, these approaches cannot be elucidated in soil-grown plants, with the exception of certain abilities with the GLO-Roots system [49]. Therefore, in parallel to the increased improvements in the phenotyping of soil-grown roots, other researchers have developed in vitro technologies to investigate root behavior under tightly regulated conditions and at high resolution. For example, the microfluidic-based root-chip is one of these approaches [50,51,52,53,54,55]. It allows precise manipulations of the root’s microenvironment with control of different stimuli, while combining stimuli, strength of stimuli, as well as symmetric and asymmetric distribution of stimuli (chemical and physical). In addition, use of microfluidic root-chip technology allows one to monitor signals at single-cell resolution, as well as inter-cellular long-distance signaling. These in vitro studies of real-time root behavior, in association with cellular signals, can be achieved using state-of-the-art microscopy [35,56]. Thus, in soil and in vitro methodologies should be considered complementary towards elucidating the mechanisms underlying root behavior and root plasticity in response to environmental cues. However, in vitro phenotyping systems should be considered with caution, particularly if roots are monitored in the light, as light shortens root length, promotes early emergence of lateral roots [57], causes the accumulation of flavonols [58], and alters the Pi starvation response [59]. Therefore, certain in vitro root phenotyping systems (e.g., D-Root) provide the conditions of cultivation in the dark [57].

## 3. Deep-Root Systems

Root system size and distribution determine access to water. In rain-fed agricultural systems, drought may be transient for short periods, or extend through the growing season, possibly leading to terminal drought, which occurs after flowering. In fact, drought is considered the most significant abiotic stress affecting crop yields in some areas, like in Mediterranean-type environments [60]. The ability of roots to reach deeper soils where water content is less variable than in shallow soils, particularly under drought conditions [60], has often been associated with drought tolerance. In different crops, quantitative trait loci (QTLs) for deep roots have been identified and found to enhance yield under drought conditions, for example, in rice, where the gene *DEEPER ROOTING 1* (*DRO1*) was cloned after being associated with a QTL for deep roots [61]. *DRO1* expression is negatively regulated by auxin and is involved in cell elongation in the root tip, which causes downward bending of the root in response to gravity. Higher expression of *DRO1* increases the root growth angle, whereby the root is in a more downward direction. Introducing *DRO1* into a shallow-rooting rice cultivar, by backcrossing, enables the resulting line to avoid drought by increasing deep rooting, which increases rice yield under drought conditions [61]. Similarly, introgression lines of wheat, in which alleles from the wild tetraploid wheat for deep roots have been introduced into hexaploidy cultivated wheat, also increased drought avoidance and improved yield under drought conditions [62]. *DRO1*-like genes prevail in diverse plant phyla, ranging from mosses to angiosperms [63,64]. They belong to the *IGT* gene family (containing the highly conserved three amino-acid motif Isoleucine, Glycine, Threonine), which also includes *Tiller Angle Control 1* (*TAC1), Negative Gravitropic Response of Roots* (*NGR),* and *LAZY1*, which are known to affect the orientation of lateral roots and shoots, and have been shown to be reasonable targets for manipulating RSA [63,64]. In wheat, the EAR-like motif IVLEM at the C-terminus of the TaADRO1-like and TaDDRO1-like is the hallmark of these proteins. TaDRO1-like interacts with TOPLESS, a repressor of auxin-regulated root-promoting genes, through the IVLEM/KLHTLIPNK motif [64]. 

**Figure 1:**
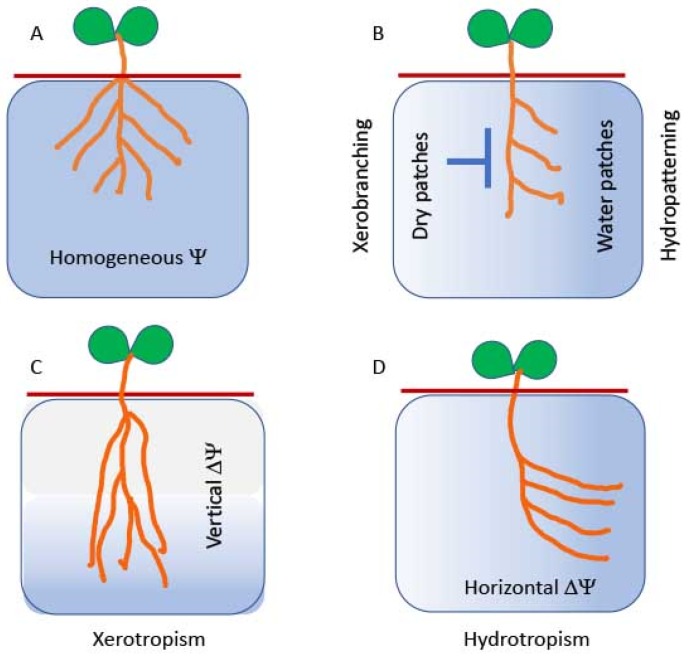
Root system architecture: Responses to water status. (**A**) Hypothetical situation of water-sufficiency and homogeneous water distribution in the soil surrounding the roots. In these conditions, the root system is likely to develop symmetrically around the root axis. (**B**) In real soil conditions, roots encounter water patches and respond by the emergence of lateral roots to the direction of the water contacts, a phenomenon termed hydropatterning [31,65], which is mediated by auxin signaling [31,35]. In contrast, roots encountering dry soil patches or air suppress lateral root formation, a phenomenon called xerobranching [32], which is mediated by abscisic acid (ABA) signaling. This is reminiscent of the inhibition of the emergence of crown roots in cereals under drought situations [40,66]. (**C**) When top soil layers are drying and deep soil layers retain sufficient water, roots may exhibit a phenomenon called xerotropism, in which the response of roots to gravity is enhanced (mediated by auxin), thus forming deeper roots with reduced angles with respect to the gravity vector [49,65]. Interestingly, xerotropism is not mediated by MIZ1 [65], which is specifically associated with hydrotropism. (**D**) Water potential asymmetry across the root may promote root curvature towards water (hydrotropism) [67,68,69,70,71]. Images of roots of soil-grown plants exhibiting xerotropism and hydropatterning can be seen in the articles cited above and in a recent review [30]. In the illustrations (A to D), darker blue represents higher water content, whereas white areas depict low water content. Ψ denotes water potential.

The ability of roots to grow deeper into soil is not only determined by the genetic makeup of the species, it is also a common response to water deficiency. This phenomenon was referred to as xerotropism [65] (Figure 1), which is defined as the tendency of plants, or plant parts, to alter their position in order to protect themselves from desiccation. Here, xerotropism involves desiccation-induced enhancement of the root gravity response, through changes in auxin biosynthesis, transport, or signaling capacity. Thus, steeper root growth angles improve water capture in several crops [40]. 

It should be noted that root penetration into the soil depends on both the root properties and soil conditions [72]. In drying soils, mechanical constraints limit root elongation. Moreover, in drying soils, the supply of water to the root may be limited and therefore, the hydrostatic pressure in root-tip cells may be insufficient to drive cell elongation underlying root elongation [72]. Moreover, the complex composition of the soil may have specific effects in different ecosystems. For example, in desert Aleppo pine forests, rocky cover and soil stoniness attenuate soil evaporation, which improves tree survival under water-limiting conditions, associated with larger soil water storage, reduced number of days under the wilting point, and consequently results in larger root systems and larger trees [73]. 

Another important function of deep roots is their involvement in a phenomenon referred to as ‘hydraulic lift’ [74,75,76]. In this process, water absorbed by deep roots moves upwards through the roots; later at night it is released in the upper soil and is stored there until it is resorbed by shallow roots the following day. Hydraulic lift is a specific case of hydraulic redistribution; it can be defined as the movement of water between soil layers with contrasting water potentials, through the plant root system. This phenomenon is most common in shrubs and trees [77,78,79]. Importantly, hydraulic lift not only provides water and nutrients to roots of a particular plant growing in shallow soil, it also provides water to neighboring plants and to root-associated microorganisms. Thus, hydraulic lift has vast implications on ecological systems, and it has also been considered for improving agricultural practices. For example, species exhibiting hydraulic lift can be combined with crops with shallow roots [80]. More recently, it was shown that hydraulic lift may even enhance surface soil nitrogen cycling and nitrogen uptake into plants [81]. Thus, deep root systems allow access to deep soil water under drought conditions and contribute to maintaining various biological activities in shallow soils.

Interestingly, cases of rapid evolution of invasive plant species have been associated with dramatic changes in root length [48]. The American-native annual plant *Heterotheca subaxillaris* (camphor-weed), which was introduced from southwest USA to the Israeli Mediterranean coastline in the 1970s for stabilizing sand dunes and preventing sand movement into agricultural areas, quickly spread, escaped its original planting areas, and has colonized extensive areas of the coastal sand dunes and beyond. Importantly, it has become a perennial plant that survives the dry summer period [48]. Comparative analysis of the roots of native USA plants with those of the invasive plant in the aeroponic growth facility at the Sarah Racine Root Research Laboratory at Tel Aviv University [46] revealed that whereas the roots of the native American plants reached a maximum of 1.5 m in length, those of the invasive plant reached lengths exceeding 5 m, and the root biomass of the latter was about three times larger than that of the former one [48]. The invasive plant also develops up to seven times more inflorescences per plant and a similar fold difference in achenes (seed) number (over 130,000 per plant). Understanding the rapid evolution of deep roots in invasive species may provide clues to improving root traits of crop plants suitable for water-limiting environments. 

## 4. Root Branching towards Water—More Is Not Necessarily Better

The phytohormone auxin functions as a positive regulator of lateral root development [82,83,84]. In recent years, using the imaging technologies described in Section 2, specific responses of roots to water and water deficiency have been reported. Apparently, plant roots use a hydropatterning mechanism to position lateral root branches toward available water [31]. This phenomenon involves auxin signaling in response to water potential differences across the roots. It was suggested that hydraulic conductivity is the key environmental variable driving this process [31]. It was also found that root growth, per se, is required for the perception of water availability to pattern root branches [85]. More recently, the signaling pathway underlying hydropatterning has been explored in greater detail. Apparently, hydropatterning is mediated by posttranslational modifications of the transcription factor ARF7 [35]. Although *ARF7* is evenly expressed around the circumferential axis of the root, it induces differential expression of its target gene *LATERAL ORGAN BOUNDARIES-DOMAIN 16* (*LBD16*) in lateral root founder cells. Namely, *LBD16* is expressed on the side of the root that is in contact with water, but not on the side that is in contact with air (dry side) [35]. This differential expression pattern of *LBD16* is regulated by differential posttranslational modification of ARF7 with the small ubiquitin-like modifier (SUMO) protein [35]. ARF7 SUMOylation negatively regulates ARF7 DNA-binding activity because SUMOylated ARF7 recruits the Aux/IAA repressor protein IAA3. Blocking ARF7 SUMOylation disrupts IAA3 recruitment and hydropatterning (on the air-exposed dry side of the root). The exact mechanism by which water potential differences across the root are translated to asymmetric cellular signals is currently unknown. What are the primary receptors of water potential differences or hydraulic conductivity, and what second messengers operate to transduce the perceived water potential differences [86,87]?

Whereas wet soil patches promote lateral root formation (hydropatterning), dry soil inhibits lateral root development, a phenomenon called xerobranching, which is mediated by ABA [32]. Similarly, drought conditions suppress shoot-borne crown roots in grasses, possibly to conserve water [66], suggesting a “water banking” scenario under water-limiting conditions. In water-limited cropping environments, saving water during vegetative growth for use during flowering and grain filling may become of great importance [88]. It was suggested that roughly 30% of the total supply of water utilized by a grain crop should be available at the time of flowering in order to produce a substantial amount of grain [88]. However, in field experiments, fewer crown roots under drought stress lead to longer crown roots that facilitate water acquisition from subsoil and thus improve drought tolerance in maize, not because of water conservation but because of a better water supply from deeper soil strata [40]. The rationale suggested [40] is that for acquiring a mobile soil resource such as water, the production of too many lateral roots is counterproductive by increasing intra-plant competition for internal resources (primarily carbohydrates) needed for root growth, as well as competition for capturing mobile soil resources, mainly water in this case [40]. Further evidence to support this hypothesis was provided by analyzing maize recombinant inbred lines under water stress in field rainout shelters, under natural drought conditions in the field. Apparently, water stress reduces lateral branching of crown roots, and lines with fewer lateral roots under stress have substantially deeper roots, greater capture of subsoil water, and consequently, improved plant water status, leaf photosynthesis, biomass, and yield [89]. Overall, plants can apparently make decisions regarding whether to conserve water for use at later stages of their life cycle, particularly at flowering and grain filling, or they can make the best of the available resources to enhance growth. Root plasticity is a major player in such decision-making.

## 5. Root Curvature Towards Water—Hydrotropism

Although hydropatterning increases root surface contact with water via developing lateral roots, and xerotropism promotes root growth toward deep soil water if there is water deficiency in the top soil layers, the typical non-homogenous water distribution in the soil surrounding the roots is sensed by the root, leading to root curvature towards the water source (sites of higher water potential). This phenomenon was described by Darwin and Darwin [67] and later termed hydrotropism [90,91], whose purpose is to place the roots near water. According to Darwin and Darwin [67], the root tip senses the water status near the root, and subsequently sends a signal to the elongation zone, where differential growth of cells on one side of the root causes it to curve towards the water source. Studies of hydrotropism revealed that ABA signaling is involved, and indeed, ABA signaling mutants exhibit attenuated hydrotropism [71]. However, ABA across the root has not been shown to form a concentration gradient or other types of asymmetric distribution corresponding to water potential differences across the root. Therefore, the specific role of ABA in hydrotropism signaling remains to be determined. 

Forward genetics approaches to isolate mutants in the hydrotropic response first revealed an ahydrotropic mutant with a point mutation in a gene named *MIZ1* (At2g41660) [68], until recently of unknown cellular or biochemical function. The mutation in *MIZ1* that abolished hydrotropism substitutes a Glycine to Glutamic acid at position 235 [68]. Another amino acid substitution in MIZ1 that abolishes hydrotropism is a Glycine to Glutamic acid change at position 203 [92]. These two Glycine residues are conserved in all 12 Arabidopsis MIZ-related proteins, which are proteins containing a DUF617 domain [68]. The *miz2* mutant that abolished hydrotropism is a weak allele of GNOM [93]. Interestingly, T-DNA insertions in the 3’ untranslated region of *MIZ1* also abolish hydrotropism [92], suggesting that RNA stability and translation play a role in *MIZ1* regulation. How MIZ2 (GNOM) is involved in hydrotropism is presently not clear. This is particularly intriguing because GNOM is well known for its role in auxin transport by regulating vesicle trafficking of PIN proteins to the membrane. However, in *Arabidopsis*, hydrotropism does not seem to be mediated by PIN trafficking [94], which is consistent with the finding that in *Arabidopsis*, in contrast to gravitropism, hydrotropism does not require auxin redistribution [95]. Moreover, in *Arabidopsis,* auxin is antagonistic to hydrotropism [95]. Thus, the relationships between the auxin transport machinery (including PIN proteins), MIZ1, MIZ2, and hydrotropism are not well understood. Contrasting effects of reactive oxygen species (ROS) on gravitropism and hydrotropism have also been described, in which ROS in root tips acts as a negative regulator of hydrotropism and a positive regulator of gravitropism [96]. ABA acts as a positive regulator of hydrotropism but as a negative regulator of gravitropism (reviewed by [79]).

**Figure 2:**
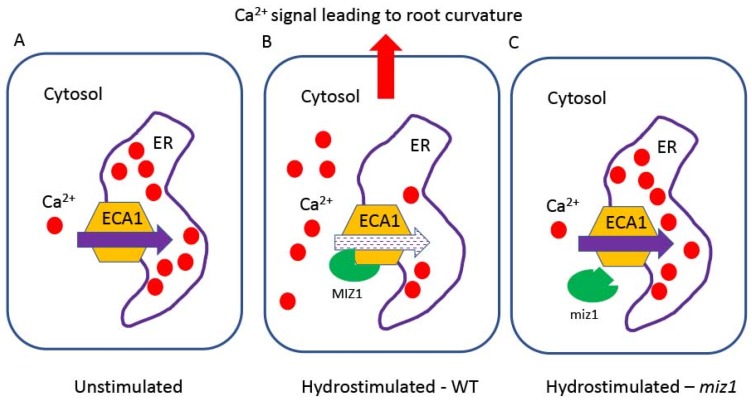
Illustration of the assumed mechanism of MIZ1-regulated generation of a cytosolic Ca^2+^ signal required for root hydrotropism [56]. MIZ1, an ER-associated protein from the side of the cytosol [97], is a negative regulator of ECA1, a Ca^2+^ efflux carrier localized in the ER. Hydrostimulation occurs when the root tip is subjected to a water potential gradient (asymmetry) across the root. (**A)** When the root is unstimulated (homogeneous distribution of water across the root tip), ECA1 is fully active and maintains a low cytosolic Ca^2+^ concentration. Under these conditions, a long-distance Ca^2+^ signal is not generated. (**B)** When the root is hydrostimulated, MIZ1 binds to ECA1 [56] and attenuates its activity. Consequently, cytosolic Ca^2+^ levels rise and an asymmetric long-distance Ca^2+^ signal is generated in the phloem. The Ca^2+^ signal peaks at the elongation zone, where differential cell elongation across the root occurs and consequently root curves towards water. (**C)** When the root is hydrostimulated in the *miz1* mutant, the mutant protein miz1 is unable to inhibit ECA1 and consequently a Ca^2+^ signal cannot be generated [56], hence root bending does not occur [56]. However, how this cellular mechanism of enhanced cytosolic Ca^2+^ in response to hydrostimulation generates a long-distance Ca^2+^ signal in the phloem [56] has yet to be determined.

According to Darwin’s hypothesis, there should be a long-distance signal from the root tip to the elongation zone. This signal should be asymmetric across the root, reflecting differences in water potentials across the root. The signal should reach the elongation zone to promote asymmetric cell elongation on opposite sides of the root to confer root curvature. Whereas Dietrich et al. [71] suggested, based on root tip ablation experiments, that the root tip is not required for the hydrotropic response, recently Shkolnik et al. [56] revealed a long-distance asymmetric Ca^2+^ signal from the root tip to the elongation zone in *Arabidopsis*, which is required for root curvature in response to water potential difference across the root [56]. The asymmetric Ca^2+^ signal that reaches the elongation zone, precedes root curvature, and is required for it to occur [56]. It remains unknown how the asymmetric Ca^2+^ signal that reaches the elongation zone is translated to differential cell elongation. Nevertheless, part of the mechanism generating the long-distance Ca^2+^ signal has been revealed [56]. Apparently, MIZ1 is a negative regulator of the endoplasmic reticulum (ER) Ca^2+^ pump ECA1, a Ca^2+^-ATPase of the Sarcoplasmic Reticulum family in animals (SERCA), which participates in maintaining cytosolic Ca^2+^ by removing it from the cytosol to the ER. In response to water potential differences across the root, inhibition of ECA1 by MIZ1 temporarily blocks this activity, which causes an increase in cytosolic Ca^2+^, underlying the long-distance signal from the root tip to the elongation zone (Figure 2). The mechanism by which an asymmetric Ca^2+^ signal is generated to reflect the asymmetric distribution of water across the root remains to be determined. The effects of ABA, Auxin, Ca^2+^ and ROS on hydrotropism and gravitropism are schematically described in [70].

Since MIZ1 is a newly discovered regulator of Ca^2+^ signaling in plants [56] and is unique to land plants [68], further structural investigations of MIZ1/ECA1 interactions may shed light on this signaling system. It would be intriguing to determine whether other MIZ-related proteins (i.e., proteins containing the DUF617 domain) in *Arabidopsis* (12 in total) are regulators of Ca^2+^ signaling and participate in tropisms, stress responses, and RSA plasticity, since several of these genes are expressed in roots (based on the Arabidopsis eFP Browser at ‘bar.utoronto.ca’ website).

Finally, it should be noted that since certain tropic responses (e.g., thigmotropism) induce the formation of a lateral root at the root bending point [98,99], which therefore contributes to RSA, it is of interest to determine if this is also the case with other tropic responses (e.g., hydrotropism). Moreover, it is of interest to determine the extent of the contribution of tropic responses to overall RSA resulting from the perception of environmental stimuli.

## 6. Acclimation to Dehydration—Root Morphology, Cellular Anatomy, Root-to-Shoot and Root-to-Root Signaling

The developmental and tropic responses described above (hydropatterning, xerotropism, and hydrotropism) facilitate plant contacts with water in the soil. However, these responses alone do not guarantee water acquisition from the soil. According to the cohesion-tension theory [100,101], water acquisition by plants is a physical process dependent on the water potential differences between soil water and those of water in the plant, and are driven mainly by the low atmospheric water potential. Therefore, this process requires: (i) Continuous water flow (either as liquid or vapor) from the soil into the root; (ii) radial movement across the root (by symplastic, transcellular, or apoplastic pathways) into the xylem water conduits; (iii) water flow up to the shoot via xylem vessels and tracheids; and (iv) water vapor flow to the atmosphere through the stomata, the key regulators of the rate of transpiration. For this to occur efficiently, anatomical, cellular, and molecular adjustments need to occur along the pathway of water movement, both in the cells comprising the radial path from the soil to the root xylem, in the xylem itself, and in aerial parts of the plant including leaves and stomata. For a background on the basic principles of this pathway, readers should consider earlier reviews [100,101]. These adjustments in anatomical, cellular, and molecular features are necessary to mitigate soil and atmosphere conditions that exacerbate drought [102]. 

A comprehensive understanding of how drought affects root traits and responses, particularly in natural ecosystems, remains elusive. The changes observed in roots of plants undergoing dehydration episodes largely depend on the duration of the exposure of the plants to dehydration, the species, other environmental conditions, and the methodology applied for analyses. Studies of trees over years of drought episodes are carried out to predict the consequences of prolonged climate change [11]. An important eco-physiological question is whether changes in root xylem and hydraulic traits result from extreme climatic events, frequency of changes, or changing average climatic conditions over time [103]. Accumulating evidence suggests that variability and extremes in climate are more important drivers of ecosystem processes than are the mean conditions. Several studies that experimentally imposed climate extremes via field experiments clearly described the negative impact of extreme drought on xylem hydraulic function and productivity [103]. Local extremes were closely linked to specific hydraulic conductivity in two Mediterranean oak species. Therefore, more frequent or more intense extreme drought events might overcome the adaptive limits of vascular transport, resulting in substantial reduction of hydraulic functionality, and hence, an increased incidence of xylem dysfunctions.

Meta-analysis of forest and woodland species from temperate and tropical regions showed a significant increase in the root-to-shoot ratio, along with a decrease in annual precipitation [11]. In another analysis, seedlings from dry forests were found to have a higher belowground biomass and deeper roots than seedlings from moist forests. These studies suggest that trees respond to water deficit by increasing their root-to-shoot ratios and rooting depth [11]. Under moderate drought, plants maintain their aboveground growth and competitiveness as long as possible. In contrast, under severe drought conditions, there is a shift to a larger root biomass with reduced shoot growth.

A more recent meta-analysis of over 120 studies under field conditions to examine the responses of 17 drought variables associated with root traits revealed that drought significantly decreases root length and root density but increases root diameter. Drought also significantly increases the root-to-shoot mass ratio. One of the most dramatic recorded changes associated with drought was an increase in root cortical aerenchyma by over 90% [104]. Another recent report concerning cereals [105] suggests that fewer axial roots, loss of parenchyma to aerenchyma, larger cortical cell size, and a reduced number of cortical cell files are more suitable for water acquisition under drought conditions. Moreover, it is important to bear in mind that root systems that are most suitable for water acquisition from deep strata would not necessarily be optimal for acquiring some minerals that are mostly present in shallow strata [105]. For example, root architectural traits that are associated with top soil foraging (e.g., for phosphorus) will have more adventitious roots, a smaller root diameter, shallower basal roots, more dispersed lateral roots, greater root biomass, longer and denser root hairs, more exudates (organic acids, protons, and phosphatases), and more mycorrhizas [105]. In contract, nitrate, which is more soluble and is found in deeper soil layers, can be acquired by deep roots.

It should also be remembered that herbaceous plants differ from woody plants that undergo extensive secondary growth [11], which in itself can respond to drought conditions. For example, the diameter of xylem conduits and the thickness of their cell walls can be modified, resulting in increased resistance against cavitation in the vascular tissues. Thus, trees have effective mechanisms to cope with dehydration that differ from those of herbaceous plants [11]. 

Other root traits associated with improved water economy in trees under drought are related to cell wall composition. For example, enhanced deposition of suberin and lignin. Suberin is a hydrophobic polymer and is an important component of endo- and exodermal cells, as well as the cork cells of the periderm in woody plants. Drought enhances the formation and deposition of root suberin, which reduces water loss from the soil and daytime transpiration. It also enhances water-use-efficiency [11]. Enhanced deposition of suberin may affect the balance between the pathways of radial water movement from the soil to the xylem and the apoplastic or cell-to-cell (symplastic and transcellular) movement. Changes in the expression of aquaporins also play a role in regulating water movement [101,106]. In the presence of heavily suberized roots, the apoplastic component of radial water flow may be too small, and the regulation of radial water flow by water channels dominates [106]. Lignin is also a major component of the vascular plant cell wall, providing mechanical support. In woody plants, drought can result in thickening and tightening of xylem tracheids, partly by the enhanced lignification of wall polymers [11]. 

Finally, acclimation to dehydration also involves long-distance communication between roots and shoots, and vice versa. Recently [107] it was reported that the *Arabidopsis* CLAVATA3/EMBRYO-SURROUNDING REGION-RELATED 25 (CLE25) peptide transmits water-deficiency signals from roots through the vascular tissues to the shoot. It affects abscisic acid biosynthesis and stomatal control of transpiration in association with BARELY ANY MERISTEM (BAM) receptors in leaves [107]. Other long-distance root-to-shoot communication may include electric signals, hydraulic signals, volatile signals and second messengers like Ca^2+^ and ROS [108]. In addition, there are indications of a root-to-root communication system, of yet unidentified nature, that may contribute to plant acclimation under water or nutrient limiting conditions [17]. Therefore, root plasticity in response to fluctuation in soil hydration is linked to shoot performance through two-way signaling processes and may also be influences by root-to-root signaling.

## 7. Plasticity of the Soil-Root-Microbe Interface—Its Relevance to Water Acquisition

In recent years, soil-root-microbe (rhizobiome) interactions have gained increasing attention and have become potential targets for improvement in order to enhance plant nutrition and resistance to both biotic and abiotic stresses including drought. Soil is considered by some researchers as the most complex biomaterial on the planet [109], and its interactions with plant roots are complex. The rhizosphere is the volume of soil adjacent to the plant roots that is affected by the roots. Root growth during cell elongation and root exudation may improve conditions for microbial colonization and pore geometry modifications of the rhizosphere by microbes [110]. There are clear differences in hydraulic properties between rhizosphere and bulk soil [37]. In addition, the abundance and function of microbes is dependent on the carbon substrates available in the rhizosphere, derived from the roots or from the breakdown of organic matter. These processes may also modify the water retention and hydraulic conductivity of the soil. However, it is not clear how rhizosphere properties and processes interact in the root zone to affect water flow towards the root system. Within the rhizosphere, there is a zone of a few millimeters of soil that closely adheres to the root system, termed the rhizosheath. This zone has strong interactions between root exudates and soil habitat, and the development of the rhizosheath itself could be viewed as a precursor to stronger interactions between roots, soil habitat, and microbial activity [110]. 

Mucigel surrounds roots [111] and is composed of compounds derived from both the roots and associated microorganisms. Mucigel, along with root hairs and fungal hyphae [19], is responsible for the agglutination of soil particles. Under conditions of water depletion, hydraulic conductivity in the soil decreases and the root may be unable to acquire sufficient water. Exudation of mucigel by the root may allow the root to form hydraulic bridges between the epidermis and the surrounding soil particles. Thus, water content may be higher near the root epidermis due to the water-holding capacity of the mucigel [112]. 

Interestingly, roots redesign their rhizosphere to alter the three-dimensional physical architecture and water dynamics [113]. These authors further suggest that breeding for rhizosheath architectures and function may be a future avenue for better designing crops in a changing environment.

The dynamic nature of the soil-root-microbe interface is affected by multiple processes, including root/microbe exudates that alter the hydrophobicity of soil pores, root cap cells sloughing from the root tip to the pore surfaces, depositing carbon sources within the rhizosphere, as well as the flow of solutes and carbon from the root, which alters conditions in the rhizosphere. Root exudation impacts the soil microbial community, influences resistance to pests, supports beneficial symbioses, alters the chemical and physical properties of the soil, and inhibits the growth of competing plant species [114]. It was estimated that the rhizosphere volume represents half of the total soil volume in a tilled surface [115]. Thus, the rhizosphere and associated rhizosheaths affect a substantial proportion of the total soil volume and functionality of the soil ecosystems.

## 8. Discussion

### 8.1. Understanding the Basis of Root Behavior and Plasticity

In their seminal book “The Power of Movement in Plants”, Darwin and Darwin [67] postulated that “the tip of the root acts like the brain of a lower animal, receiving impressions from the sense organs, and directing the several movements”. Later, J.C. Bose [116] suggested that plants and animals have essentially the same fundamental physiological mechanisms, and that plants coordinate their movements and responses to the environment through electrical signaling. Bose further suggested that all plants are sensitive explorers of their world, responding to it through a fundamental, pulsatile motif involving coupled oscillations in electric potential, turgor pressure, contractility, and growth (reviewed by [117]). Although several aspects of Darwin’s ‘root-brain’ hypothesis remain controversial, as recently discussed [118], it is widely accepted that plants perform sophisticated information processing and computation, which rely on learning and memory [119,120,121,122,123,124,125]. These features are common to plants and animals, and underlie their adaptation to the continuously changing environment [126,127]. A major challenge emerging from Darwin’s hypothesis (often referred to as the metaphoric ‘Root-Brain’ hypothesis) is to understand how the different stimuli are perceived and processed by the root tip, leading to “decisions” that underlie root behavior, including, for example: (i) Direction of growth (curvature and angle relative the gravity vector); (ii) the extent of growth (acceleration or arrest); (iii) root branching (the formation of lateral or adventitious roots); (iv) the emergence of root hairs; and (v) other anatomical, morphological, and metabolic adjustments. These behavioral aspects rely on coordinated cell divisions and elongation of various root cell types, and on communication between cells [124]. The outcome of the continuous interactions between the plant’s pre-disposed developmental program, inherent in the genome, and the ever-changing environment is the establishment of a 3D root architecture that reflects the tremendous plasticity of root development. However, to date, knowledge about the mechanisms underlying root behavior and the responses to its environment is fragmented. It is believed that no single cell makes decisions on behalf of an organ [124,126]. Rather, organ-scale decision-making occurs in a distributed fashion and emerges from the collective states of individual cells, similar to neurons in animals [119,123,124]. Moreover, roots are probably not pre-programmed to respond to all of the almost infinite possible microenvironments. Rather, there are probably certain genetically dictated rules under which cells, tissues, and organs integrate the multiple signals originating from interactions with their environment. 

Molecular genetics, combined with advanced imaging technologies of both soil-grown plants [30] and in vitro-grown plants using state-of-the-art microscopy [35,56], now enable to study root behavior in greater detail, revealing root responses to environmental cues including their pursuit of water. However, several fundamental questions remain unanswered: How do roots respond to multiple stimuli that are perceived simultaneously? How do roots quantify different environmental stimuli? How are pre-determined response hierarchies (e.g., water deficiency overcomes gravity) integrated into the root’s environmental information processing system? How do roots respond to symmetrically versus asymmetrically distributed stimuli? And, how much of the 3D environment surrounding the root does a root actually sense and respond to? Answers to these questions require the ability to analyze and image roots and signals within the roots on a three-dimensional level and at different scales of cellular organizations, ideally ranging from subcellular organization, to single cells, tissues, whole organ, and whole plant. Such studies should be combined with cell-specific whole-genome ‘omics’ and analyzed using a system biology approach and computational modeling [128,129,130]. In addition, the molecular mechanisms underlying signal perception and transduction need to be elucidated down to their atomic level structures, because sensing and transducing cellular signals may be explained by specific atomic-level changes within a single molecule, or within a complex of molecules. Finally, as technologies improve and, consequently, more data are obtained, mathematical modeling and machine-learning approaches [131] must be applied to elucidate the principles underlying root behavior and their interaction with the environment. In this respect, plant biologists may benefit from earlier studies in other systems and previously described decision-making theories [132]. Finally, understanding root behavior in response to environmental cues may help predict how climate change affects plant ecology and, at the same time, provide tools for crop breeding by manipulating root plasticity and optimizing root development and proliferation. 

### 8.2. Targeting Root Traits for Crop Improvement

The first ‘green revolution’ in the mid 20th century relied on improving cereal crops for higher yields by increasing the use of fertilizers and by identifying dwarf varieties that did not lodge. It has now become increasingly clear that further agricultural developments are needed to enhance food production by 70% by 2050 [1]. This requires a ‘second green revolution’ [105] that should aim at increasing yields and product quality along with reducing agricultural inputs and environmental impacts, while facing the challenges of both biotic and abiotic threats associated with increasing climate change. It was suggested that the highest potential of increasing global crop yields lies in marginal lands and in developing countries [1]. Moreover, the challenge of maintaining and improving yields with low water supply and quality will be critical, and increased tolerance of crops to drought (and salinity) is needed [1,39]. Therefore, root traits that improve water acquisition are major targets for genetic manipulation. 

Giehl and von Wirén [133] suggested that from the perspective of water acquisition, breeding crops with a predefined root system architecture may be less appropriate than exploiting plasticity and sensing mechanisms to improve root adaptability to spatial and temporal variations in soil moisture. In this context, manipulating the molecular mechanism and tapping into possible natural allelic variations in hydropatterning, xerotropism, and hydrotropism have potential for breeding crops that are better able to withstand environmental stresses. 

Success in identifying genes that underlie root traits associated with drought tolerance and water acquisition is promising for developing crops better suited for agriculture under low and less stable water supplies. Improved genomic tools that can now be applied to numerous plant species, including major crops, with fast-developing root phenotyping systems in vitro, in soil, and in the field, together with bioinformatics and computational biology tools, provide the basis for better understanding root behavior and for developing crops more suitable for a sustainable agriculture. The aim is to achieve higher yields and better quality, but with reduced agricultural inputs and environmental footprint. 

However, to succeed, these advanced plant science tools and advanced technologies must be integrated into other agricultural management disciplines [134], while considering human cultural diversity [135] and specific and often diverse needs in developing countries. If we are to build a sustainable and desirable future, we need to be able to understand, model, and value complex social-cultural-agricultural-ecological systems in a comprehensive way [136]. 

## References

[1] Tester M., Langridge P. (2010). Breeding technologies to increase crop production in a changing world. Science.

[2] Feulner G. (2017). Global challenges: Climate change. Glob. Chall..

[3] Ray D.K., Gerber J.S., MacDonald G.K., West P.C. (2015). Climate variation explains a third of global crop yield variability. Nat. Commun..

[4] Comas L.H., Becker S.R., Cruz V.M., Byrne P.F., Dierig D.A. (2013). Root traits contributing to plant productivity under drought. Front. Plant Sci..

[5] Zhao M., Running S.W. (2010). Drought-induced reduction in global terrestrial net primary production from 2000 through 2009. Science.

[6] Blum A. (2017). Osmotic adjustment is a prime drought stress adaptive engine in support of plant production. Plant Cell Environ..

[7] Sade N., Gebremedhin A., Moshelion M. (2012). Risk-taking plants: Anisohydric behavior as a stress-resistance trait. Plant Signal. Behav..

[8] Yoo C.Y., Pence H.E., Jin J.B., Miura K., Gosney M.J., Hasegawa P.M., Mickelbart M.V. (2010). The Arabidopsis GTL1 transcription factor regulates water use efficiency and drought tolerance by modulating stomatal density via transrepression of SDD1. Plant Cell.

[9] Yoo C.Y., Mano N., Finkler A., Weng H., Day I.S., Reddy A.S.N., Poovaiah B.W., Fromm H., Hasegawa P.M., Mickelbart M.V. (2019). A Ca^2+^/CaM-regulated transcriptional switch modulates stomatal development in response to water deficit. Sci. Rep..

[10] Giarola V., Hou Q., Bartels D. (2017). Angiosperm plant desiccation tolerance: Hints from transcriptomics and genome sequencing. Trends Plant Sci..

[11] Brunner I., Herzog C., Dawes M.A., Arend M., Sperisen C. (2015). How tree roots respond to drought. Front. Plant Sci..

[12] Nicotra A.B., Atkin O.K., Bonser S.P., Davidson A.M., Finnegan E.J., Mathesius U., Poot P., Purugganan M.D., Richards C.L., Valladares F. (2010). Plant phenotypic plasticity in a changing climate. Trends Plant Sci..

[13] Lobet G., Paez-Garcia A., Schneider H., Junker A., Atkinson J.A., Tracy S. (2019). Demystifying roots: A need for clarification and extended concepts in root phenotyping. Plant Sci..

[14] Taiz L., Zeiger E., Moller I.M., Murphy A. (2015). Plant Physiology and Development.

[15] Bellini C., Pacurar D.I., Perrone I. (2014). Adventitious roots and lateral roots: Similarities and differences. Annu. Rev. Plant Biol..

[16] Meister R., Rajani M.S., Ruzicka D., Schachtman D.P. (2014). Challenges of modifying root traits in crops for agriculture. Trends Plant Sci..

[17] Falik O., Mordoch Y., Ben-Natan D., Vanunu M., Goldstein O., Novoplansky A. (2012). Plant responsiveness to root-root communication of stress cues. Ann. Bot..

[18] Haling R.E., Brown L.K., Bengough A.G., Young I.M., Hallett P.D., White P.J., George T.S. (2013). Root hairs improve root penetration, root-soil contact, and phosphorus acquisition in soils of different strength. J. Exp. Bot..

[19] Moreno-Espindola I.P., Rivera-Becerril F., Ferrara-Guerrero M.J., De Leon-Gonzalez F. (2007). Role of root-hairs and hyphae in adhesion of sand particles. Soil Biol. Biochem..

[20] Tanaka N., Kato M., Tomioka R., Kurata R., Fukao Y., Aoyama T., Maeshima M. (2014). Characteristics of a root hair-less line of Arabidopsis thaliana under physiological stresses. J. Exp. Bot..

[21] Kwasniewski M., Daszkowska-Golec A., Janiak A., Chwialkowska K., Nowakowska U., Sablok G., Szarejko I. (2016). Transcriptome analysis reveals the role of the root hairs as environmental sensors to maintain plant functions under water-deficiency conditions. J. Exp. Bot..

[22] Miguel M.A., Postma J.A., Lynch J.P. (2015). Phene synergism between root hair length and basal root growth angle for phosphorus acquisition. Plant Physiol..

[23] York L.M., Nord E.A., Lynch J.P. (2013). Integration of root phenes for soil resource acquisition. Front. Plant Sci..

[24] Lynch J.P., Chimungu J.G., Brown K.M. (2014). Root anatomical phenes associated with water acquisition from drying soil: Targets for crop improvement. J. Exp. Bot..

[25] York L.M., Lynch J.P. (2015). Intensive field phenotyping of maize (*Zea mays* L.) root crowns identifies phenes and phene integration associated with plant growth and nitrogen acquisition. J. Exp. Bot..

[26] Lynch J.P. (2019). Root phenotypes for improved nutrient capture: An underexploited opportunity for global agriculture. New Phytol..

[27] Schmidt J.E., Gaudin A.C.M. (2017). Toward an integrated root ideotype for irrigated systems. Trends Plant Sci..

[28] Lynch J.P. (2013). Steep, cheap and deep: An ideotype to optimize water and N acquisition by maize root systems. Ann. Bot..

[29] Cantarel A.A., Pommier T., Desclos-Theveniau M., Diquélou S., Dumont M., Grassein F., Kastl E.M., Grigulis K., Laîné P., Lavorel S. (2015). Using plant traits to explain plant–microbe relationships involved in nitrogen acquisition. Ecology.

[30] Morris E.C., Griffiths M., Golebiowska A., Mairhofer S., Burr-Hersey J., Goh T., von Wangenheim D., Atkinson B., Sturrock C.J., Lynch J.P. (2017). Shaping 3D Root System Architecture. Curr. Biol..

[31] Bao Y., Aggarwal P., Robbins N.E., Sturrock C.J., Thompson M.C., Tan H.Q., Tham C., Duan L., Rodriguez P.L., Vernoux T. (2014). Plant roots use a patterning mechanism to position lateral root branches toward available water. Proc. Natl. Acad. Sci. USA.

[32] Orman-Ligeza B., Morris E.C., Parizot B., Lavigne T., Babé A., Ligeza A., Klein S., Sturrock C., Xuan W., Novák O. (2018). The Xerobranching Response Represses Lateral Root Formation When Roots Are Not in Contact with Water. Curr. Biol..

[33] Giri J., Bhosale R., Huang G., Pandey B.K., Parker H., Zappala S., Yang J., Dievart A., Bureau C., Ljung K. (2018). Rice auxin influx carrier OsAUX1 facilitates root hair elongation in response to low external phosphate. Nat. Commun..

[34] Huang G., Liang W., Sturrock C.J., Pandey B.K., Giri J., Mairhofer S., Wang D., Muller L., Tan H., York L.M. (2018). Rice actin binding protein RMD controls crown root angle in response to external phosphate. Nat. Commun..

[35] Orosa-Puente B., Leftley N., von Wangenheim D., Banda J., Srivastava A.K., Hill K., Truskina J., Bhosale R., Morris E., Srivastava M. (2018). Root branching toward water involves posttranslational modification of transcription factor ARF7. Science.

[36] Segal R., Kushnir T., Mualem Y., Shani U. (2017). Microsensing of Water Dynamics and Root Distributions in Sandy Soils. Vadose Zone J..

[37] Daly K.R., Mooney S.J., Bennett M.J., Crout N.M., Roose T., Tracy S.R. (2015). Assessing the influence of the rhizosphere on soil hydraulic properties using X-ray computed tomography and numerical modelling. Exp. Bot..

[38] Daly K.R., Tracy S.R., Crout N.M.J., Mairhofer S., Pridmore T.P., Mooney S.J., Roose T. (2018). Quantification of root water uptake in soil using X-ray computed tomography and image-based modelling. Plant Cell Environ..

[39] Tuberosa R. (2012). Phenotyping for drought tolerance of crops in the genomics era. Front. Physiol..

[40] Lynch J.P. (2018). Rightsizing root phenotypes for drought resistance. J. Exp. Bot..

[41] Bucksch A., Burridge J., York L.M., Das A., Nord E., Weitz J.S., Lynch J.P. (2014). Image-based high-throughput field phenotyping of crop roots. Plant Physiol..

[42] Das A., Schneider H., Burridge J., Ascanio A.K., Wojciechowski T., Topp C.N., Lynch J.P., Weitz J.S., Bucksch A. (2015). Digital imaging of root traits (DIRT): A high-throughput computing and collaboration platform for field-based root phenomics. Plant Methods.

[43] Postma J.A., Kuppe C., Owen M.R., Mellor N., Griffiths M., Bennett M.J., Lynch J.P., Watt M. (2017). OpenSimRoot: Widening the scope and application of root architectural models. New Phytol..

[44] Zhu X.G., Lynch J.P., LeBauer D.S., Millar A.J., Stitt M., Long S.P. (2016). Plants in silico: Why, why now and what? An integrative platform for plant systems biology research. Plant Cell Environ..

[45] Marshall-Colon A., Long S.P., Allen D.K., Allen G., Beard D.A., Benes B., von Caemmerer S., Christensen A.J., Cox D.J., Hart J.C. (2017). In Silico: Generating Virtual Crops Using an Integrative and Multi-scale Modeling Platform. Front. Plant Sci..

[46] Waisel Y., Waisel Y., Eshel A., Kafkafi U. (2002). Aeroponics: A tool for root research under minimal environmental restrictions. Plan Roots: The Hidden Half.

[47] Eshel A., Grünzweig J.M. (2013). Root-shoot allometry of tropical forest trees determined in a large-scale aeroponic system. Ann. Bot..

[48] Sternberg M. (2016). From America to the holy land: Disentangling plant traits of the invasive *Heterotheca subaxillaris* (Lam.) Britton & Rusby. Plant Ecol..

[49] Rellan-Alvarez R., Lobet G., Dinneny J.R. (2016). Environmental control of root system biology. Annu. Rev. Plant Biol..

[50] Nezhad A.S. (2014). Microfluidic platforms for plant cells studies. R. Soc. Chem..

[51] Elitas M., Yuce M., Budak H. (2017). Microfabricated tools for quantitative plant biology. Analyst.

[52] Jiang H., Wang X., Nolan T.M., Yin Y., Aluru M.R., Dong L. Automated microfluidic plant chips-based plant phenotyping system. Proceedings of the 12th IEEE International Conference on Nano and Micro Engineered and Molecular Systems.

[53] Stanley C.E., Grossmann G., Solvas X.C., DeMello A.J. (2016). Soil-on-a-Chip: Microfluidic platforms for environmental organismal studies. Lab Chip.

[54] Stanley C.E., Shrivastava J., Brugman R., Heinzelmann E., van Swaay D., Grossmann G. (2018). Dual-flow-RootChip reveals local adaptations of roots towards environmental asymmetry at the physiological and genetic levels. New Phytol..

[55] Massalha H., Korenblum E., Malitsky S., Shapiro O., Aharoni A. (2017). Live imaging of root-bacteria interactions in a microfluidic set-up. Proc. Natl. Acad. Sci. USA.

[56] Shkolnik D., Nuriel R., Bonza M.C., Costa A., Fromm H. (2018). MIZ1 regulates ECA1 to generate a slow, long-distance phloem-transmitted Ca^2+^ signal essential for root water tracking in Arabidopsis. Proc. Natl. Acad. Sci. USA.

[57] Silva-Navas J., Moreno-Risueno M.A., Manzano C., Pallero-Baena M., Navarro-Neila S., Téllez-Robledo B., Garcia-Mina J.M., Baigorri R., Gallego F.J., del Pozo J.C. (2015). D-Root: A system for cultivating plants with the roots in darkness or under different light conditions. Plant J..

[58] Silva-Navas J., Moreno-Risueno M.A., Manzano C., Téllez-Robledo B., Navarro-Neila S., Carrasco V., Pollmann S., Gallego F.J., Del Pozo J.C. (2016). Flavonols Mediate Root Phototropism and Growth through Regulation of Proliferation-to-Differentiation Transition. Plant Cell.

[59] Silva-Navas J., Conesa C.M., Saez A., Navarro-Neila S., Garcia-Mina J.M., Zamarreño A.M., Baigorri R., Swarup R., Del Pozo J.C. (2019). Role of cis-zeatin in root responses to phosphate starvation. New Phytol..

[60] Saradadevi R., Palta J.A., Siddique K.H.M. (2017). ABA-Mediated Stomatal Response in Regulating Water Use during the Development of Terminal Drought in Wheat. Front. Plant Sci..

[61] Uga Y., Sugimoto K., Ogawa S., Rane J., Ishitani M., Hara N., Kitomi Y., Inukai Y., Ono K., Kanno N. (2013). Control of root system architecture by DEEPER ROOTING 1 increases rice yield under drought conditions. Nat. Genet..

[62] Merchuk-Ovnat L., Fahima T., Ephrat J.E., Krugman T., Saranga Y. (2017). Ancestral QTL alleles from wild emmer what enhance root development under drought in modern wheat. Front. Plant Sci..

[63] Guseman J.M., Webb K., Srinivasan C., Dardick C. (2017). DRO1 influences root system architecture in Arabidopsis and Prunus species. Plant J..

[64] Ashraf A., Rehman O.U., Muzammil S., Léon J., Naz A.A., Rasool F., Ali G.M., Zafar Y., Khan M.R. (2019). Evolution of Deeper Rooting 1-like homoeologs in wheat entails the C-terminus mutations as well as gain and loss of auxin response elements. PLoS ONE.

[65] Feng W., Lindner H., Robbins N.E., Dinneny J.R. (2016). Growing Out of Stress: The Role of Cell-and Organ-Scale Growth Control in Plant Water-Stress Responses. Plant Cell.

[66] Sebastian J., Yee M.C., Goudinho Viana W., Rellán-Álvarez R., Feldman M., Priest H.D., Trontin C., Lee T., Jiang H., Baxter I. (2016). Grasses suppress shoot-borne roots to conserve water during drought. Proc. Natl. Acad. Sci. USA.

[67] Darwin C., Darwin F. (1880). The Power of Mmovement in Plants.

[68] Kobayashi A., Takahashi A., Kakimoto Y., Miyazawa Y., Fujii N., Higashitani A., Takahashi H. (2007). A gene essential for hydrotropism in roots. Proc. Natl. Acad. Sci. USA.

[69] Eapen D., Barroso M.L., Ponce G., Campos M.E., Cassab G.I. (2005). Hydrotropism: Root growth responses to water. Trends Plant Sci..

[70] Shkolnik D., Fromm H. (2016). The Cholodny-Went theory does not explain hydrotropism. Plant Sci..

[71] Dietrich D., Pang L., Kobayashi A., Fozard J.A., Boudolf V., Bhosale R., Antoni R., Nguyen T., Hiratsuka S., Fujii N. (2017). Root hydrotropism is controlled via a cortex-specific growth mechanism. Nat. Plants.

[72] Jin K., Shen J., Ashton R.W., Dodd I.C., Parry M.A., Whalley W.R. (2013). How do roots elongate in a structured soil?. J. Exp. Bot..

[73] Preisler Y., Tatarinov F., Grünzweig J.M., Bert D., Ogée J., Wingate L., Rotenberg E., Rohatyn S., Her N., Moshe I. (2019). Mortality versus survival in drought-affected Aleppo pine forest depends on the extent of rock cover and soil stoniness. Funct. Ecol..

[74] Caldwell M.M., Richards J.H. (1989). Hydraulic lift: Water efflux from upper roots improves effectiveness of water uptake by deep roots. Oecologia.

[75] Caldwell M.M., Dawson T.E., Richards J.H. (1998). Hydraulic lift: Consequences of water efflux from the roots of plants. Oecologia.

[76] Dawson T.E. (1993). Hydraulic lift and water use by plants: Implications for water balance, performance and plant-plant interactions. Oecologia.

[77] Sekiya N., Yano K. (2002). Water acquisition from rainfall and groundwater by legume crops developing deep rooting systems determined with stable hydrogen isotope compositions of xylem water. Field Crop. Res..

[78] Sekiya N., Yano K. (2004). Do pigeon pea and sesbania supply groundwater to intercropped maize through hydraulic lift?—Hydrogen stable isotop inestigation of xylem waters. Field Crop. Res..

[79] Prieto I., Ryel R.J. (2014). Internal hydraulic redistribution prevents the loss of root conductivity during drought. Tree Phyisiol..

[80] White J.C., Liste H.H. (2008). Plant hydraulic lift of soil water—Implications for crop production and land restoration. Plant Soil.

[81] Cardon Z.G., Stark J.M., Herron P.M., Rasmussen J.A. (2013). Sagebrush carring out hydraulic lift enhances surface soil nitrogen cycling and nitrogen uptake into inflorescences. Proc. Natl. Acad. Sci. USA.

[82] Porco S., Larrieu A., Du Y., Gaudinier A., Goh T., Swarup K., Swarup R., Kuempers B., Bishopp A., Lavenus J. (2016). Lateral root emergence in Arabidopsis is dependent on transcription factor LBD29 regulation of auxin influx carrier LAX3. Development.

[83] Swarup K., Benková E., Swarup R., Casimiro I., Péret B., Yang Y., Parry G., Nielsen E., De Smet I., Vanneste S. (2008). The auxin influx carrier LAX3 promotes lateral root emergence. Nat. Cell Biol..

[84] Lavenus J., Goh T., Roberts I., Guyomarc’h S., Lucas M., De Smet I., Fukaki H., Beeckman T., Bennett M., Laplaze L. (2013). Lateral root development in Arabidopsis: Fifty shades of auxin. Trends Plant Sci..

[85] Robbins N.E., Dinneny J.R. (2018). Growth is required for perception of water availability to pattern root branches in plants. Proc. Natl. Acad. Sci. USA.

[86] Scharwies J.D., Dinneny J.R. (2019). Water transport, perception, and response in plants. J. Plant Res..

[87] Fromm H., Fichman Y., Sopory S. (2019). Water sensing in plants. Sensory Biology of Plants.

[88] Ahmed M.A., Passioura J., Carminati A. (2018). Hydraulic processes in roots and the rhizosphere pertinent to increasing yield of water-limited grain crops: A critical review. J. Exp. Bot..

[89] Zhan A., Schneider H., Lynch J.P. (2015). Reduced lateral root branching density improves drought tolerance in maize. Plant Physiol..

[90] Von Sachs J. (1887). Relations between growth and cell-division in the embryonic tissues. Lecture XXVII. Lectures on the Physiology of Plants.

[91] Jaffe M.J., Takahashi H., Biro R.L. (1985). A pea mutant for the study of hydrotropism in roots. Science.

[92] Sherman T. (2013). Characterizing Genes and Mechanisms Involved in Root Hydrotropism in Arabidopsis Thaliana. Ph.D. Thesis.

[93] Miyazawa Y., Takahashi A., Kobayashi A., Kaneyasu T., Fujii N., Takahashi H. (2009). GNOM-mediated vesicular trafficking plays an essential role in hydrotropism of Arabidopsis roots. Plant Physiol..

[94] Moriwaki T., Miyazawa Y., Fujii N., Takahashi H. (2014). GNOM regulates root hydrotropism and phototropism independently of PIN-mediated auxin transport. Plant Sci..

[95] Shkolnik D., Krieger G., Nuriel R., Fromm H. (2016). Hydrotropism: Root Bending Does Not Require Auxin Redistribution. Mol. Plant.

[96] Krieger G., Shkolnik D., Miller G., Fromm H. (2016). Reactive Oxygen Species Tune Root Tropic Responses. Plant Physiol..

[97] Yamazaki T., Miyazawa Y., Kobayashi A., Moriwaki T., Fujii N., Takahashi H. (2012). MIZ1, an essential protein for root hydrotropism, is associated with the cytoplasmic face of the endoplasmic reticulum membrane in Arabidopsis root cells. FEBS Lett..

[98] Ditengou F.A., Teale W.D., Kochersperger P., Flittner K.A., Kneuper I., van der Graaff E., Nziengui H., Pinosa F., Li X., Nitschke R. (2008). Mechanical induction of lateral root initiation in Arabidopsis thaliana. Proc. Natl. Acad. Sci. USA.

[99] Richter G.L., Monshausen G.B., Krol A., Gilroy S. (2009). Mechanical stimuli modulate lateral root organogenesis. Plant Physiol..

[100] Steudle E. (2001). The cohesion-tension mechanism and the acquisition of water by plant roots. Annu. Rev. Plant. Physiol. Plant Mol. Biol..

[101] Williams M., Oliver M., Pallardy S. (2014). Water Relations 1: Uptake and Transport. Teaching tools in plant biology: Lecture notes. Plant Cell.

[102] Bramley H., Turner N.C., Turner D.W., Tyerman S.D. (2009). Roles of morphology, anatomy, and aquaporins in determining contrasting hydraulic behavior of roots. Plant Physiol..

[103] Rita A., Borghetti M., Todaro L., Saracino A. (2016). Interpreting the climatic effects on xylem functional traits in two Mediterranean oak species: The role of extreme climatic events. Front. Plant Sci..

[104] Zhou G., Zhou X., Nie Y., Bai S.H., Zhou L., Shao J., Cheng W., Wang J., Hu F., Fu Y. (2018). Drought-induced changes in root biomass largely result from altered root morphological traits: Evidence from a synthesis of global field trials. Plant Cell Environ..

[105] Lynch J.P. (2007). Roots of the second green revolution. Aust. J. Bot..

[106] Steudle E. (2000). Water uptake by roots: Effects of water deficit. J. Exp. Bot..

[107] Takahashi F., Suzuki T., Osakabe Y., Betsuyaku S., Kondo Y., Dohmae N., Fukuda H., Yamaguchi-Shinozaki K., Shinozaki K. (2018). A small peptide modulates stomatal control via abscisic acid in long-distance signalling. Nature.

[108] Choi W.G., Hilleary R., Swanson S.J., Kim S.H., Gilroy S. (2016). Rapid, Long-Distance Electrical and Calcium Signaling in Plants. Annu. Rev. Plant Biol..

[109] Young I.M., Crawford J.W. (2004). Interactions and self-organization in the soil-microbe complex. Science.

[110] Rabbi S.M.F., Tighe M.K., Knox O., Young I.M. (2018). The impact of carbon addition on the organisation of rhizosheath of chickpea. Sci. Rep..

[111] Jenny H., Grossenbacher K. (1963). Root-soil boundary ones as seen in the electron microscope. Soil Sci. Soc. Am. J..

[112] York L.M., Carminati A., Mooney S.J., Ritz K., Bennett M.J. (2016). The holistic rhizosphere: Integrating zones, processes, and semantics in the soil influenced by roots. J. Exp. Bot..

[113] Rabbi S.M.F., Tighe M.K., Flavel R.J., Kaiser B.N., Guppy C.N., Zhang X., Young I.M. (2018). Plant roots redesign the rhizosphere to alter the three-dimensional physical architecture and water dynamics. New Phytol..

[114] Bertin C., Yang X., Leslie A., Weston L.A. (2003). The role of root exudates and allelochemicals in the rhizosphere. Plant Soil.

[115] Bengough A.G. (2012). Water dynamics of the root zone: Rhizosphere biophysics and its control of soil hydrology. Vadose Zone J..

[116] Bose J.C. (1926). The Nervous Mechanism of Plants. Nature.

[117] Shepherd V.A. (2005). From semi-conductors to the rhythms of sensitive plants: The research of J.C. Bose. Cell Mol. Biol..

[118] Taiz L., Alkon D., Draguhn A., Murphy A., Blatt M., Hawes C., Thiel G., Robinson D.G. (2019). Plants neither possess nor require consciousness. Trends Plant Sci..

[119] Peak D., West J.D., Messinger S.M., Mott K.A. (2004). Evidence for complex, collective dynamics and emergent, distributed computation in plants. Proc. Natl. Acad. Sci. USA.

[120] Baluska F., Levin M. (2016). On having no head: Cognition throughout biological systems. Front. Psychol..

[121] Trewavas A. (2003). Aspects of plant intelligence. Ann. Bot..

[122] Trewavas A. (2016). Intelligence, Cognition, and Language of Green Plants. Front. Psychol..

[123] Topham A.T., Taylor R.E., Yan D., Nambara E., Johnston I.G., Bassel G.W. (2017). Temperature variability is integrated by a spatially embedded decision-making center to break dormancy in Arabidopsis seeds. Proc. Natl. Acad. Sci. USA.

[124] Bassel G.W. (2018). Information processing and distributed computation in plant organs. Trends Plant Sci..

[125] Chamovitz D.A. (2018). Plants are intelligent; now what?. Nat. Plants.

[126] Trewavas A.J. (2015). Plant Behavior and Intelligence.

[127] Trewavas A.J. (2017). The foundations of plant intelligence. Interface Focus.

[128] Libault M., Pingault L., Zogli P., Schiefelbein J. (2017). Plant systems biology at the single-cell level. Trends Plant Sci..

[129] Rutten J.P., Ten Tusscher K. (2019). In Silico Roots: Room for Growth. Trends Plant Sci..

[130] Santos Teixeira J.A., Ten Tusscher K.H. (2019). The Systems Biology of Lateral Root Formation: Connecting the Dots. Mol. Plant.

[131] Nicholas J.A., Herbert Chan H.W. (2018). Baker MAB Machine learning: Applications of artificial intelligence to imaging and diagnosis. Biophys. Rev..

[132] Conradt L. (2012). Models in animal collective decision-making: Information uncertainty and conflicting preferences. Interface Focus.

[133] Giehl R.F.H., von Wirén N. (2018). Hydropatterning-how roots test the waters. Science.

[134] Haro von Mogel K. (2013). Interactions key to beating future droughts. CSA News.

[135] Adger W.N., Barnett J., Brown K., Marshall N., O’Brien K. (2013). Cultural dimensions of climate change impacts and adaptation. Nat. Clim. Chang..

[136] Nicholls R.J., Craig W., Hutton C.W., Adger W.N., Hanson S.E., Rahman M.M., Salehin M. (2018). Ecosystem Services for Well-Being in Deltas, Integrated Assessment for Policy Analysis.

